# Supplementary figures and images for: Effective mechanical potential of cell–cell interaction explains three-dimensional morphologies during early embryogenesis
Source: PLoS Comput Biol. 2023 Aug 7;19(8):e1011306. doi: 10.1371/journal.pcbi.1011306 (PMC10434874; doi:10.1371/journal.pcbi.1011306)

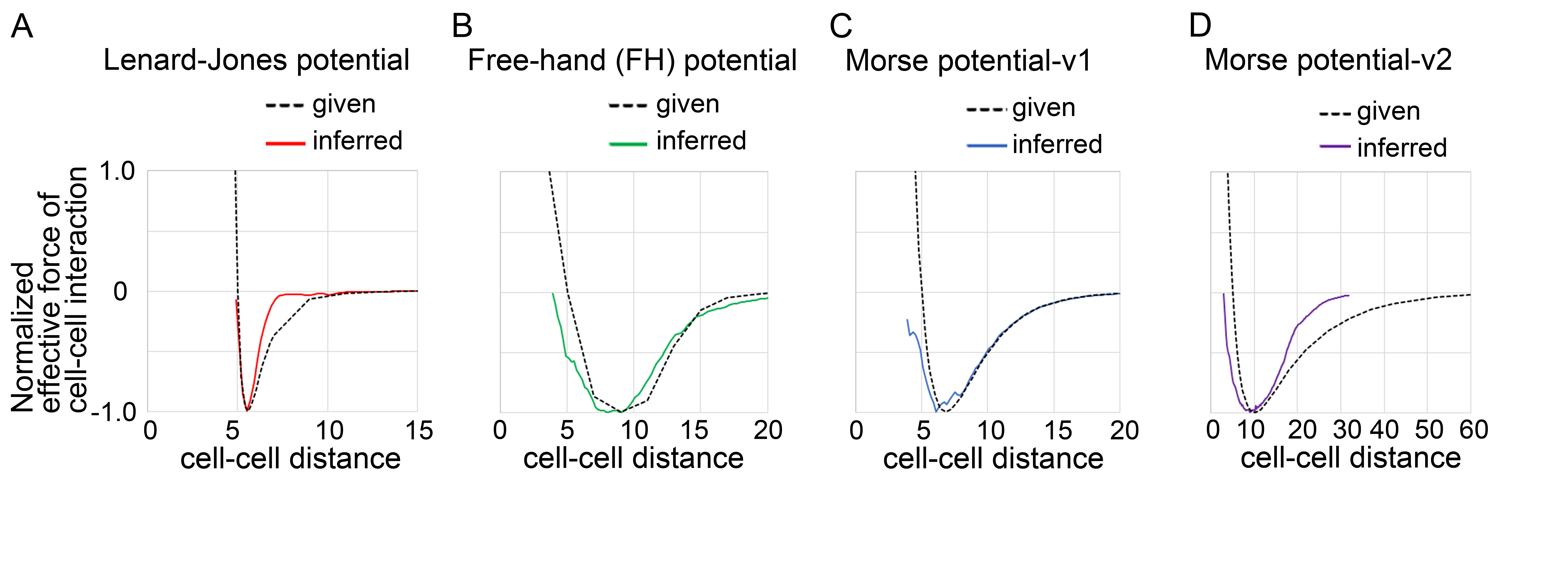

Supplement: S3 Fig — Validation of force inference method for various potentials. Distance–force (DF) curves were inferred from simulation data generated under various potentials. The force values were normalized by the maximum attractive forces in the given potential. The mean cell diameters were set to be 5.0, where the forces are 0. The given potentials are the LJ (A), FH (B), and Morse (C and D) from previous two papers [18,20]. Solid lines, inferred DF curves; broken lines, the given potentials. (TIF) [file pcbi.1011306.s004.tif]

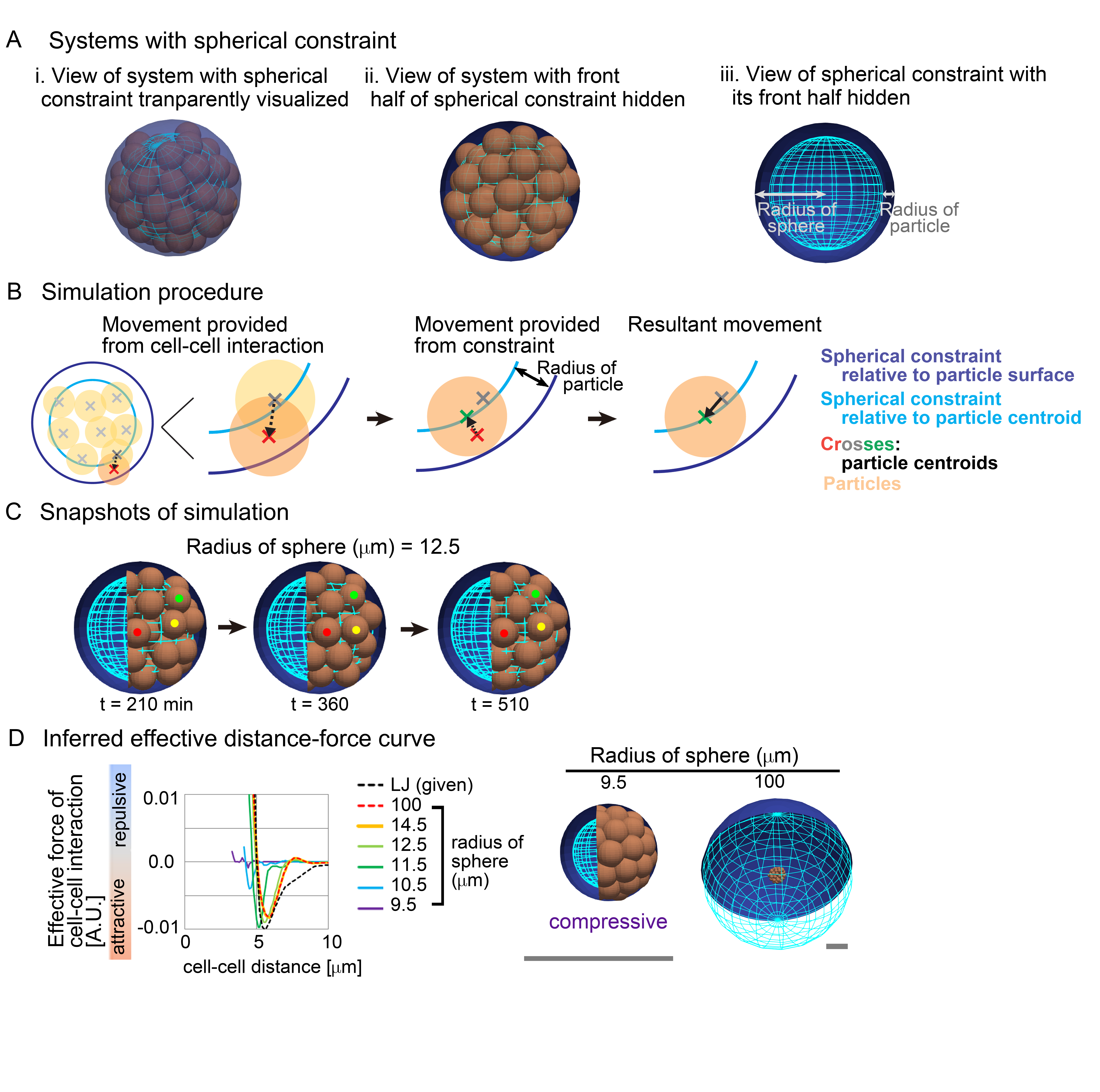

Supplement: S4 Fig — Inference of effective forces under spherical constraints. Simulation data were used to validate our inference method. Systems with spherical constraints were considered. A. Particles were embedded into a spherical constraint (A-i and -ii). Definitions of the radius of the sphere and the radius of particles are shown (A-iii). B. The simulation procedures when a particle collides with the spherical constraint are described (dark orange circle). The detailed procedures are described in S1 Text (Section 6-2-2). C. Snapshots of simulations. The Lenard-Jones potential (LJ) was provided. Three particles are marked by red, green, and yellow. The number of particles were set to be 64 in all conditions. The condition is [sampling interval = 3.0 min, SD value of force fluctuation = 1000, and persistency of force fluctuation = 1 min]. D. Inferred DF curves under different radius of spheres. Two snapshots under the different radius are shown. In the case that 9.5μm, the particles were very closely contacted each other so that the distances between the adjacent particles seemed to be less than the diameter of the particles. Therefore, each particle was compressed. In the case that the radius was 100μm, the spherical constraint was sufficiently large so that the particles are not in contact with the surface of the constraint. The black scale bars and the gray ones correspond to the diameter of the particles and the diameter of the spherical constraint with the radius = 14.5μm. (TIF) [file pcbi.1011306.s005.tif]

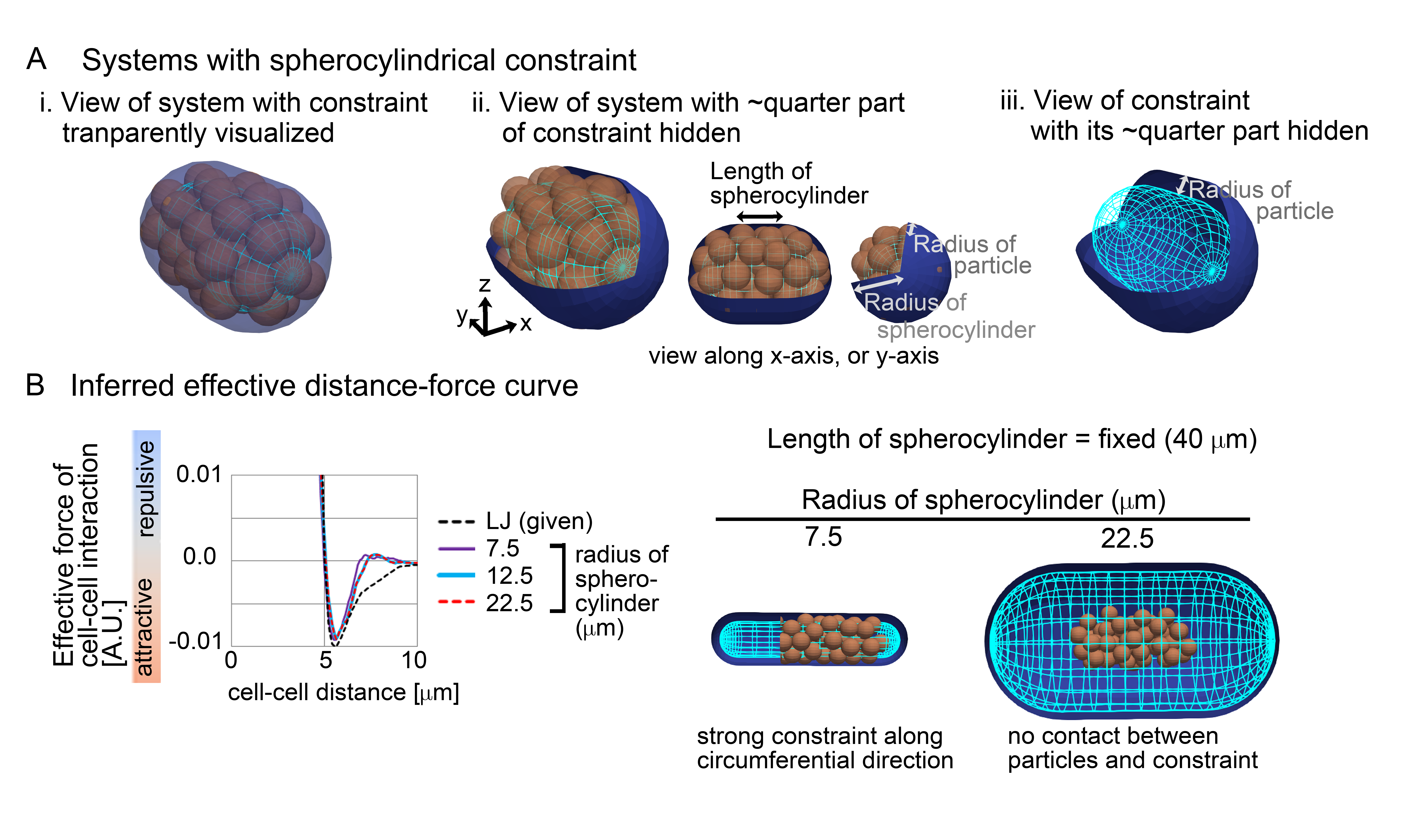

Supplement: S5 Fig — Inference of effective forces under spherocylindrical constraints. Simulation data were used to validate our inference method. Systems with spherocylindrical constraints were implemented. A. Particles were embedded into a spherocylindrical constraint (A-i and -ii). Definitions of the radius and the length of the spherocylinder, and the radius of particles are shown (A-ii and -iii). The simulation procedures when a particle collides with the spherocylindrical constraint were implemented in a similar manner to S4 Fig. B. Inferred DF curves under different radius of the spherocylindrical constraints. Two snapshots under the different radius are shown. The particles are in close contact with the surface of the constraint in the case that the radius was 7.5μm. In the case that the radius was 22.5μm, the spherocylindrical constraint was sufficiently large so that the particles are not in contact with the surface of the constraint. The number of particles were set to be 54 in all conditions. The condition is [sampling interval = 3.0 min, SD value of force fluctuation = 1000, and persistency of force fluctuation = 1 min]. (TIF) [file pcbi.1011306.s006.tif]

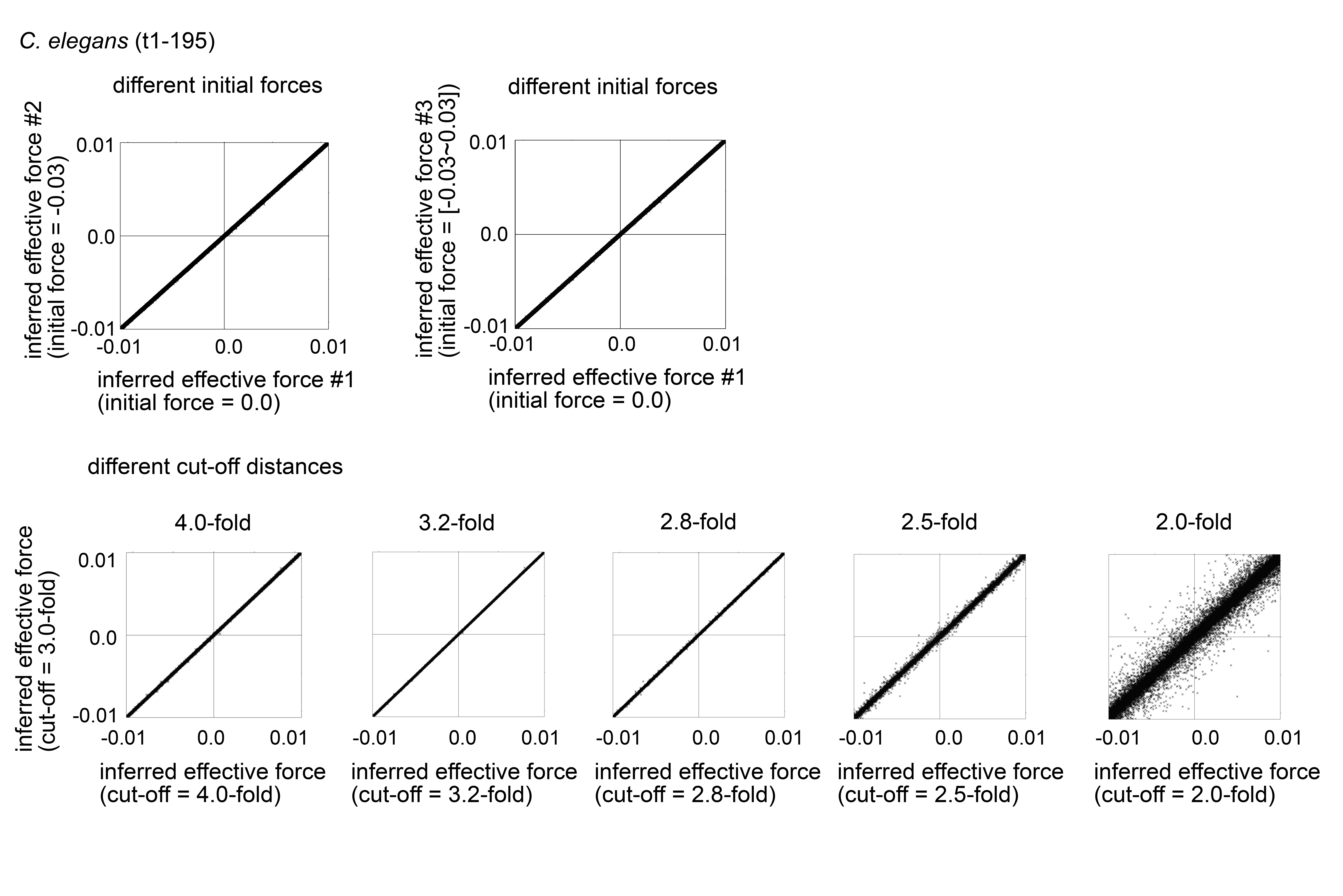

Supplement: S6 Fig — Uniqueness of solutions of effective force inference in C. elegans. In the top left panel, the minimizations of Equation S6 were performed from different initial force values as described in the x- and y- axes. In the top right panel, the initial forces were given as uniform random numbers ranging from -0.03 to 0.03 in the y-axis. The inferred values of each cell-cell interaction were plotted by crosses. The inferred values from the different initial force values were absolutely correlated in the all cases, suggesting that a unique solution was obtained in each system. In the bottom panels, different cut-off distances were given as indicated (3.0, 4.0, 3.2, 2.8, 2.5 and 2.0-fold diameter of cell bodies), and the minimizations were performed. The inferred values were strongly correlated when the cut-off distances were greater than 2.8-fold diameter of cell bodies, suggesting that a unique solution was obtained under these conditions. (TIF) [file pcbi.1011306.s007.tif]

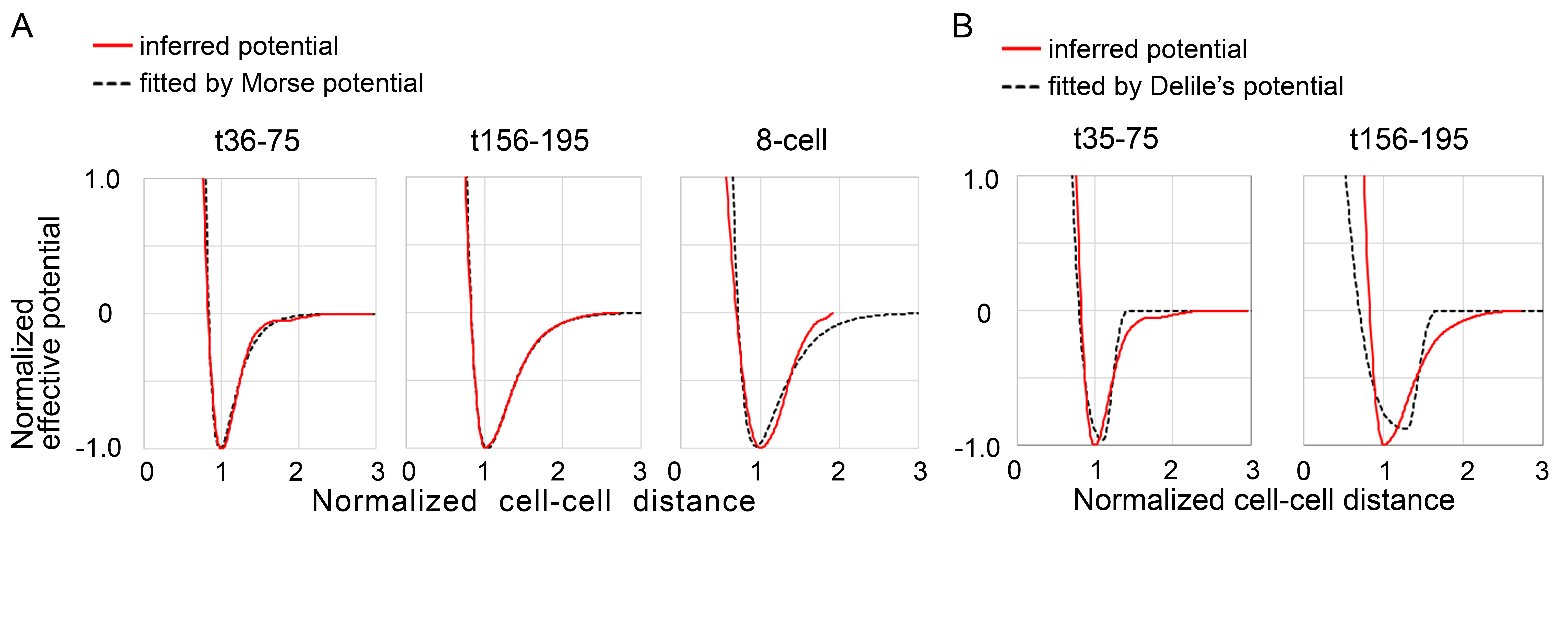

Supplement: S7 Fig — Fitting of the previously-reported potentials to inferred distance–potential (DP) curves in C. elegans and mouse embryos. A. The Morse potential was used for fitting. The formula of the Morse potential is: U(D)=Ue[exp{−2a(D−De)}−2exp{−a(D−De)}], where U is the potential energy, D is the particle–particle distance, and Ue, De, and a are the fitting parameters. Cell–cell distances were normalized by the distances providing the potential minima in the inferred potentials. Effective potential energies were normalized so that the potential minima become -1.0. t36-75 and t156-195 are from the C. elegans as defined in Fig 4, and 8-cell is from the mouse 8-cell stage embryo. Fitting was performed by using the solver implemented in the Excel software. B. A potential proposed by Delile et al. was used for fitting [18]. The distance–force curve was described in the Delile’s paper, from which we numerically computed the DP curve for fitting. (TIF) [file pcbi.1011306.s008.tif]

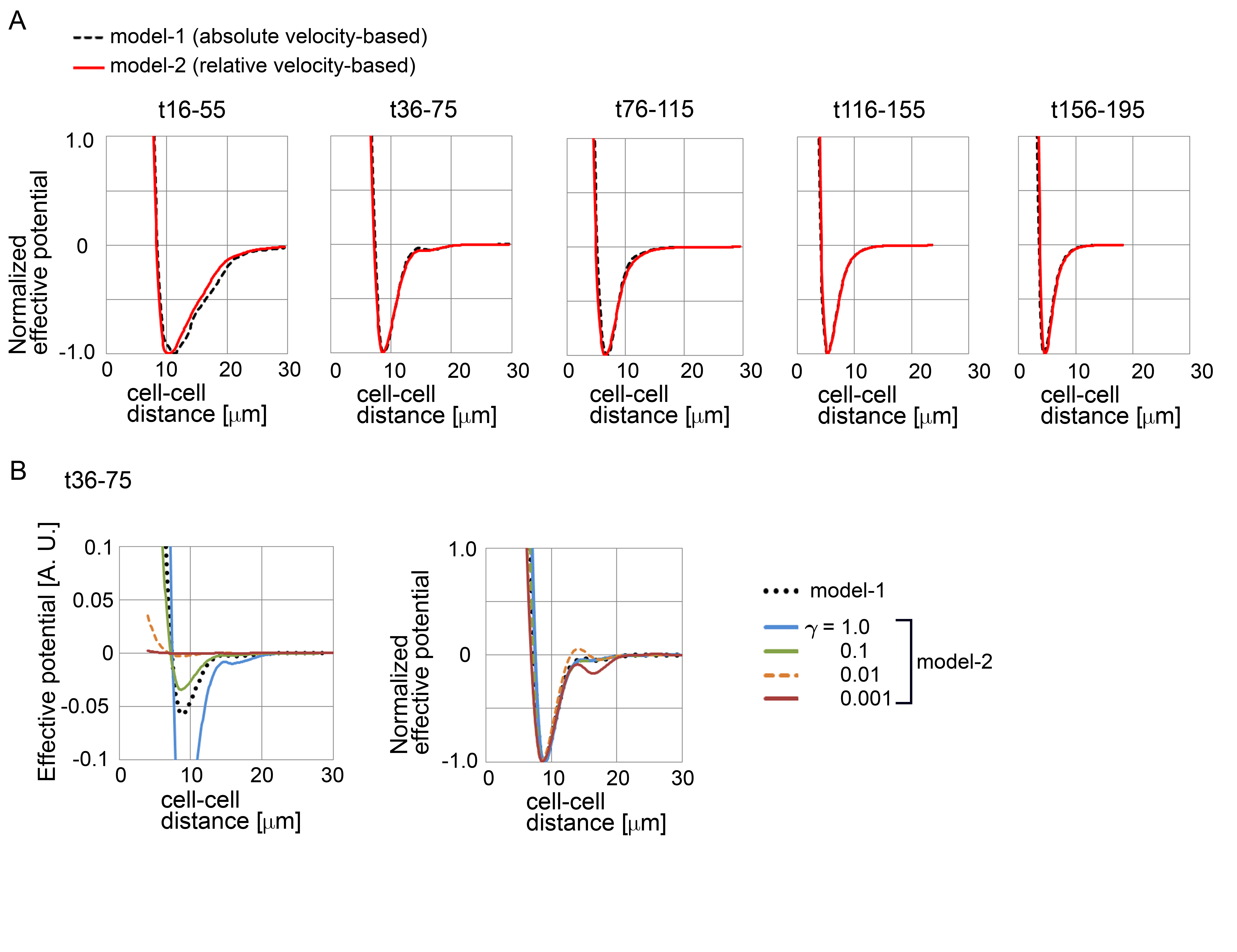

Supplement: S8 Fig — Inferred distance–potential (DP) curves under the assumption of the relative velocity-dependent model (Eq S1-2) in C. elegans embryos. A. Comparison of the inferred DP curves under the two models: the absolute velocity-based and the relative velocity-based models. The effective potential energies were normalized so that the potential minima become -1.0. The time frames (e.g., t16-55, etc) were defined in Fig 4. B. Parameter dependency of inferred DP curves in C. elegans embryo at the t36-75 time frame. The values of γ in Equation S1-2 were variously set. The inferred DP curve from the absolute velocity-based model (model-1) is also presented for comparison, where γ was set 1.0. In the relative velocity-based model, because ωg(Dpm) in the Equation S2-1 was set to be 1.0 at Dpm = the diameters of cell bodies, the values of {ωg(Dpm)γ} at Dpm = the diameters of cell bodies are equal to γ. (TIF) [file pcbi.1011306.s009.tif]

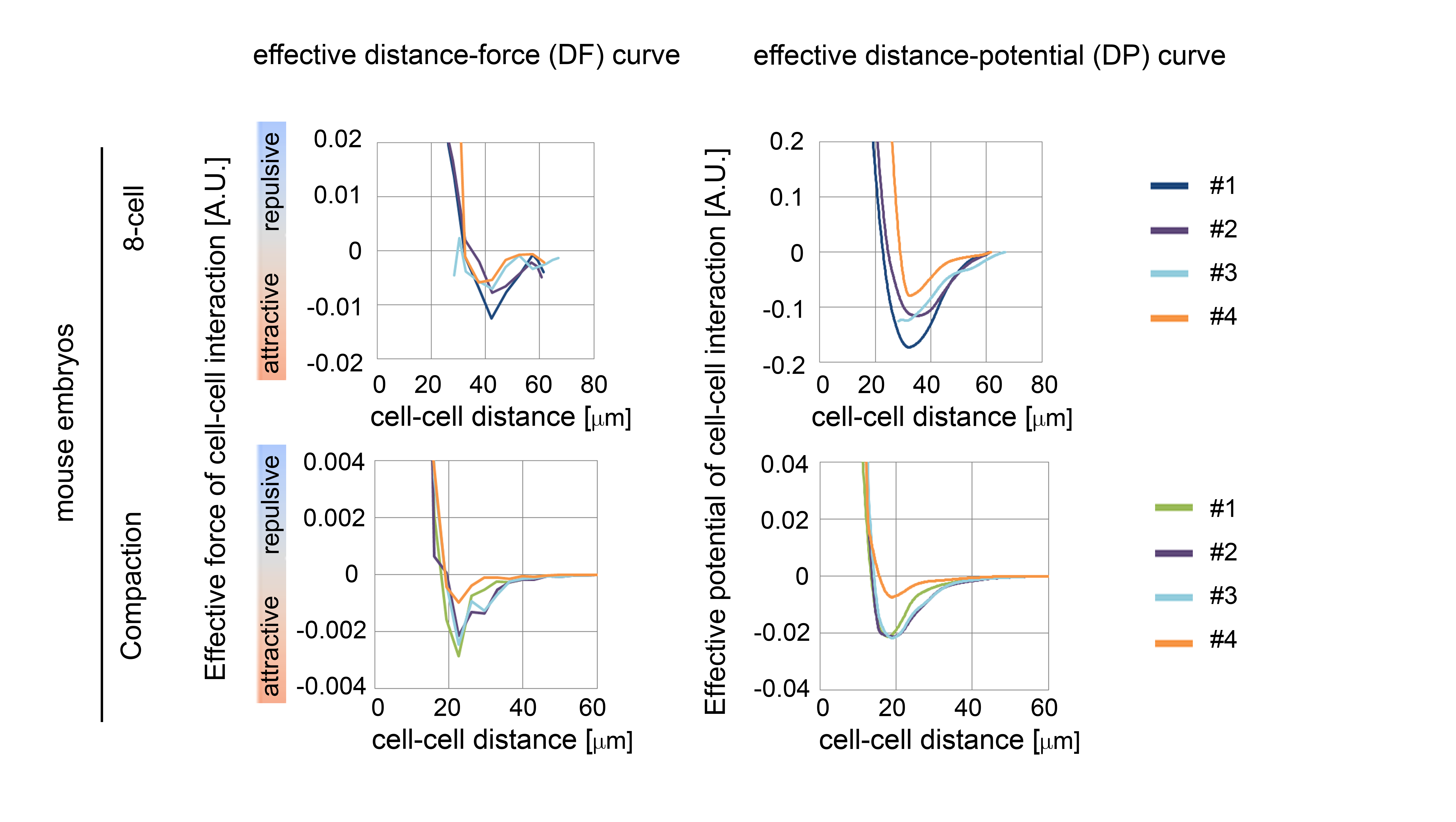

Supplement: S9 Fig — Distance–force and distance–potential curves in mouse embryos. Inferred DF and DP curves in mouse 8-cell and compaction stages. Four independent embryos (#1–4) were analyzed for each stage. #1 for each stage corresponds to Fig 5. (TIF) [file pcbi.1011306.s010.tif]

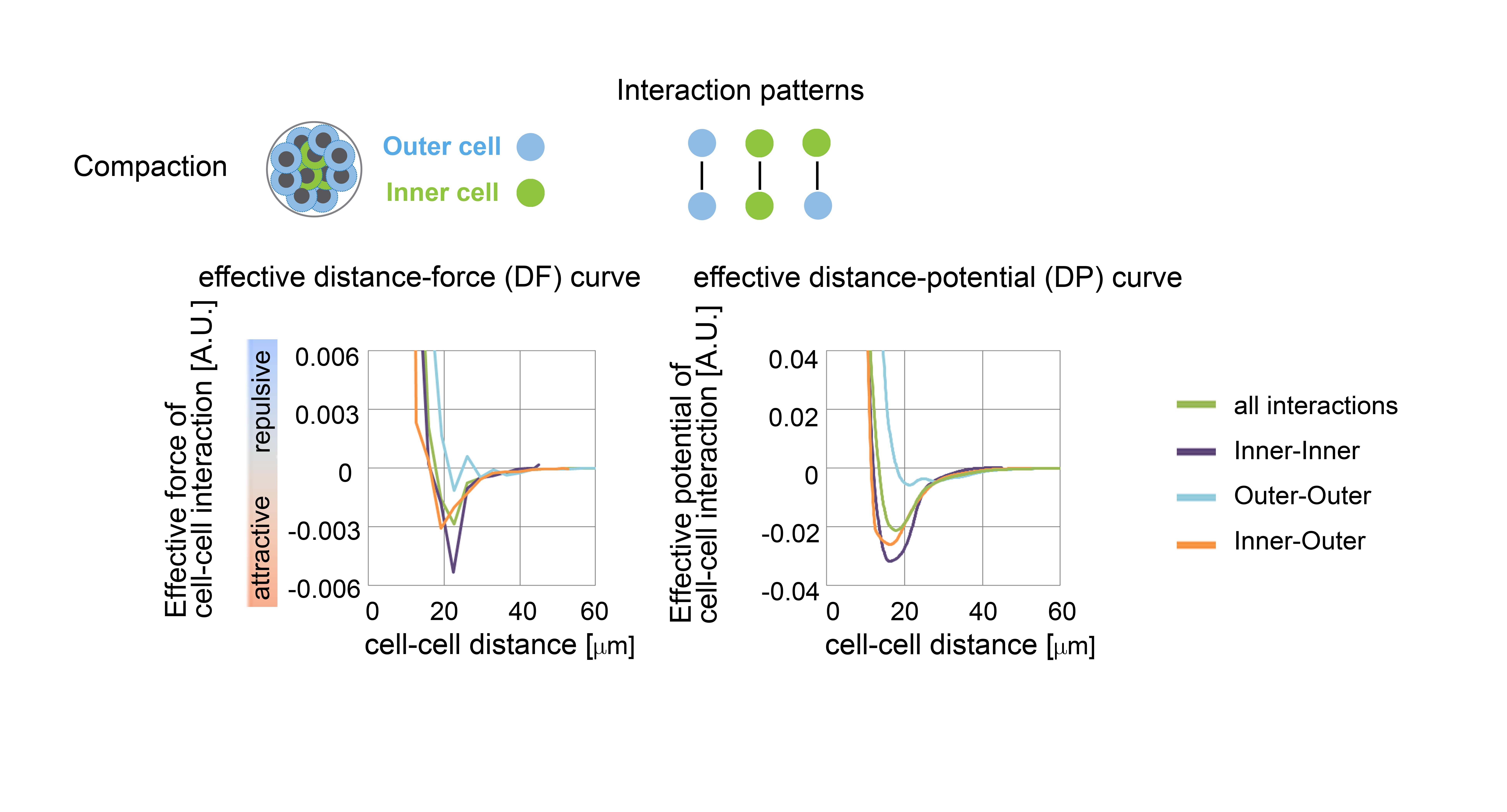

Supplement: S10 Fig — Distance–force and distance–potential curves of different cell types in mouse embryos. A. Inferred DF and DP curves of outer (blue circles) and inner (green circles) cells in mouse compaction stage. There are three possible interactions: inner–inner, outer–outer, and inner–outer cells. These data were obtained from embryo #1 in S9 Fig, compaction. The curves of all interactions (green lines) are identical to those in S9 Fig. (TIF) [file pcbi.1011306.s011.tif]

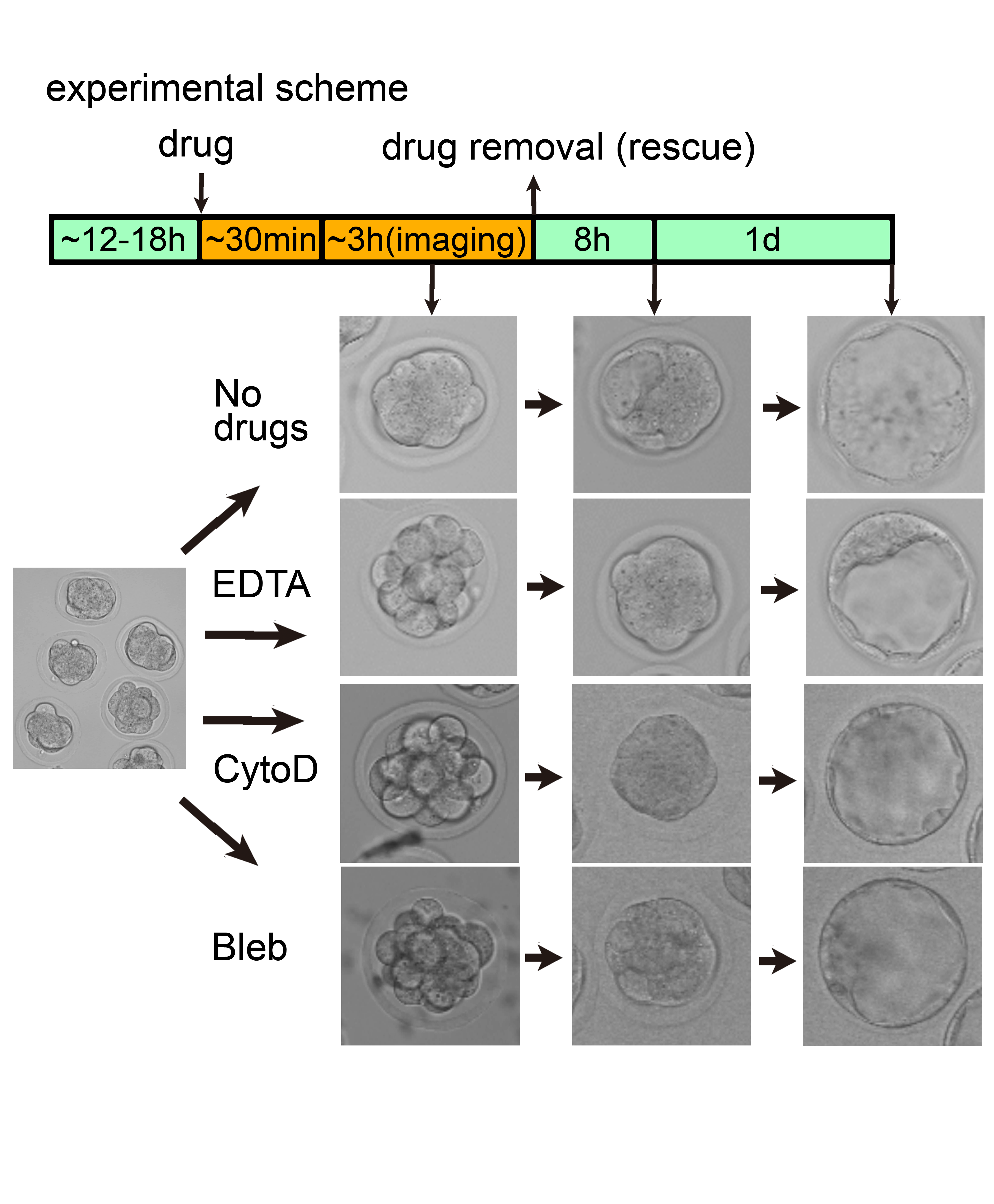

Supplement: S11 Fig — Experimental design for inhibiting compaction in mouse embryos. The experimental design of Fig 7 is shown. The upper panel is the experimental scheme. The lower panels are microscopic images of embryos at each step of the experimental scheme. CytoD, cytochalasin D; Bleb, blebbistatin. (TIF) [file pcbi.1011306.s012.tif]

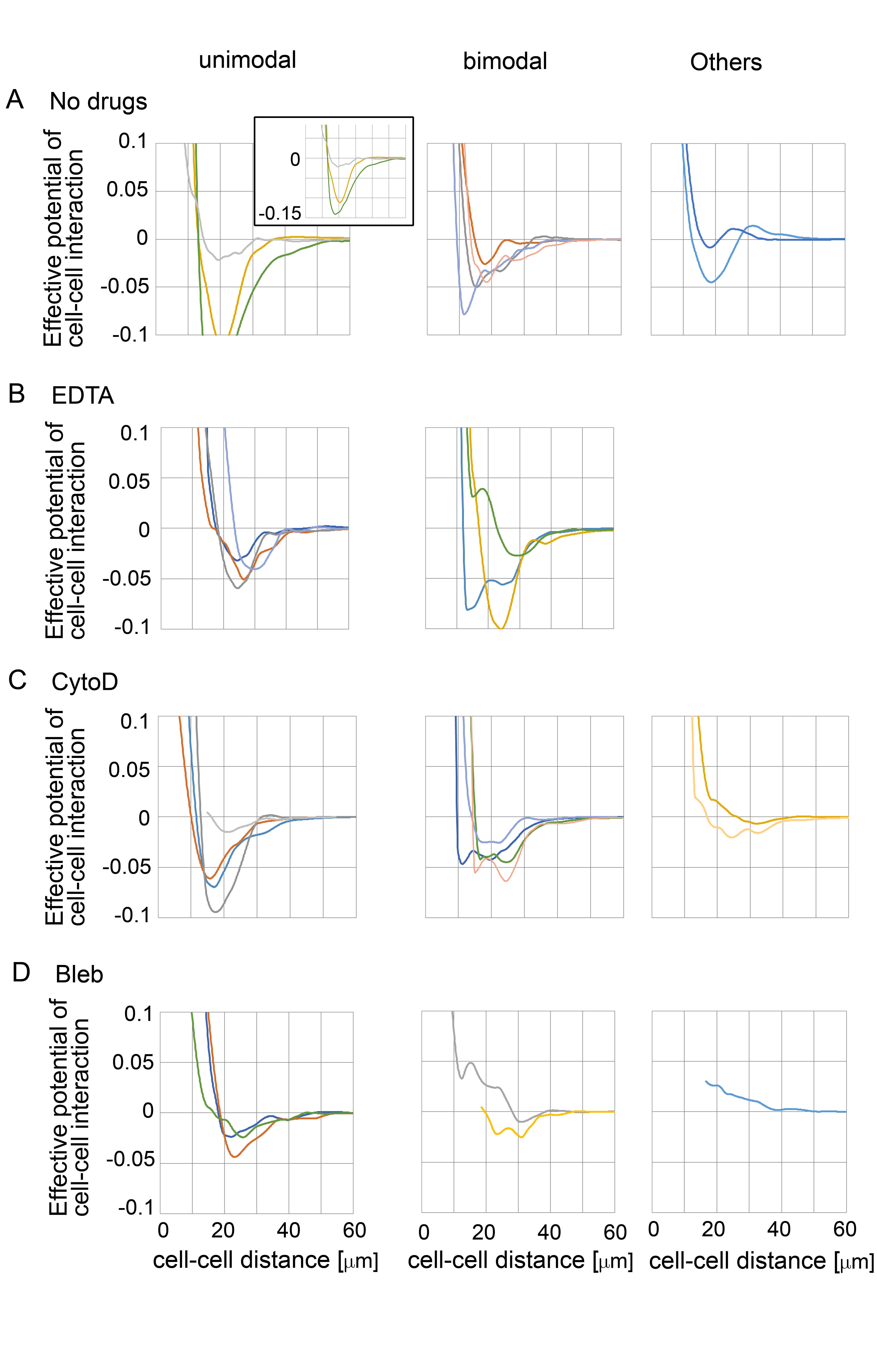

Supplement: S12 Fig — Distance–potential curve in compaction-inhibited mouse embryos. The DP curves in Fig 7 are enlarged. For visualization, the DP curves were roughly categorized into three groups; unimodal (almost single potential minimum), bimodal (almost double potential minima), and others (the distance at potential minimum is very long, or a potential maximum exists). CytoD, cytochalasin D; Bleb, blebbistatin. (TIF) [file pcbi.1011306.s013.tif]

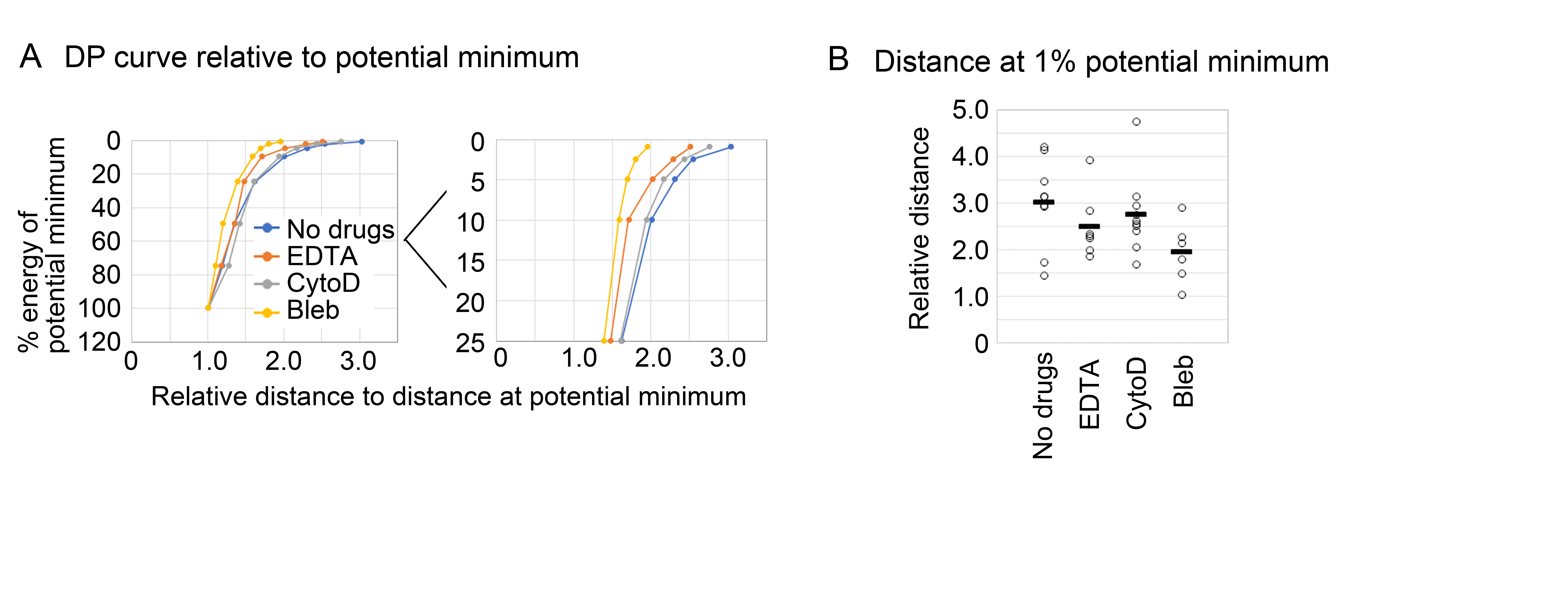

Supplement: S14 Fig — Quantitative comparison of profiles of distance–potential curves in compaction-inhibited mouse embryos. A. Related to the right panel of Fig 7C. Distances at given % of potential minima were calculated as the relative distances to the distances at the potential minima. 75, 50, 25, 10, 5, 2.5, and 1% were considered. The mean values were plotted. CytoD, cytochalasin D; Bleb, blebbistatin. B. The results under 1% in A. Mann–Whitney–Wilcoxon tests were performed and the resultant p-values for “No Drugs” vs. “EDTA”, vs. “CytoD”, and vs. “Bleb” are 0.21, 0.21, and 0.036, respectively. In the case for 10% potential minima (Fig 7C, right panel), the p-values are 0.30, 0.60, and 0.11. Black bar, mean. (TIF) [file pcbi.1011306.s015.tif]
