# Supplementary material for: Effective mechanical potential of cell–cell interaction explains three-dimensional morphologies during early embryogenesis
Source: PLoS Comput Biol. 2023 Aug 7;19(8):e1011306. doi: 10.1371/journal.pcbi.1011306 (PMC10434874; doi:10.1371/journal.pcbi.1011306)
Supplement: Attachment — Submitted filename: Response_to_reviewers230510v2_forSubumit.pdf [file pcbi.1011306.s024.pdf]

Title: Effective mechanical potential of cell–cell interaction explains three-dimensional morphologies during early embryogenesis

Authors: Koyama et al.
PCOMPBIOL-D-22-01824

We appreciate the reviewer's fruitful comments. We have revised our manuscript with a few new data which would substantially strengthen our manuscript. We have highlighted major revised parts in the main text by yellow.

Point-by-point responses to reviewer's comments

Reviewer #1

*****Minor comment-1: "Line 125: I don't think the statement that pairwise interactions between cells have never been measured is correct. There definitely are experiments that have looked at the pairwise interaction potentials between cells in atomic microscopy settings. Having said that, I agree with the authors general assertion that such profiles have not been inferred in multicellular systems (as done in this study)."

***Response to the minor comment-1

We agree with the reviewer's comment. We have revised our manuscript as the reviewer pointed out.

> Line 130; "In multicellular systems, the pairwise potentials are conceptually well-known [7,8,11,12]. But the profiles have been never measured nor inferred in multicellular systems,…"

*****Minor comment-2: "Line 213: Is the role of F_i^2 essentially to force a minimization of forces between cell pairs?"

***Response to the minor comment-2

Yes, that is right. We think that, in our original manuscript, the meaning of the cost function containing F_i^2 was obscure. We have revised our manuscript as follows.

> Line 227; "…where F_i is the force value of *i*th cell–cell interactions,…"
>
> Line 231; "…, leading to that the value of F_i approaches zero especially around larger D_i…"

*****Minor comment-3: "Fig 2: The inferred potentials can clearly match the potential dip. However, it appears that in all the scenarios the inferred potential increases faster to 0 after the dip. Is there a reason for this? Could this be caused by crowding? i.e. that piece of the potential cannot be inferred because due to crowding i.e. cell pairs of that separation are not observed in the data?"

***Response to the minor comment-3

As pointed out by the reviewer, the inferred potentials from synthetic data shows a faster decay to 0 from minus values in Fig. 2B and 2C, compared with the Lenard-Jones (LJ) potential by which these

synthetic data were generated. We agree with the reviewer's idea that this is caused by crowding. Indeed, in the contrast to Fig. 2B and 2C, in Fig. 2A where particles are assembling from relatively sparse distributions, the decay was equivalent to that of the LJ potential. Under crowded situations, there are many cells adjacent to one cell. When inferring cell–cell interaction forces, cell movements may be reproduced just by adjacent cell–cell interactions without considering long cell–cell interactions. Therefore, forces around long cell–cell interactions given by the LJ potential are easily masked. This is one limitation of our method. We do not know that this limitation becomes critical in real (in vivo) systems. But we speculate that, under crowded situations, long cell–cell interactions hardly occur, resulting in non-effective.

> Line 272; "In addition, around longer cell–cell distances, the inferred DF curves showed slight inconsistencies with the LJ potential in the case of Fig. 2B and 2C but not 2A. This may be caused by crowding, because the particles in Fig. 2A were sparsely distributed compared with Fig. 2B and 2C."

*****Minor comment-4: "Line 267: I generally agree with the statement. However, there are maybe two points worth discussing or mentioning:

1. Cell volumes can change. Some cells feature aquaporins which facilitate the transport of water between the extracellular and intracellular space.
2. Many particle models (for instance those by Drasdo et. al for the growth of tumour spheroids) rely on cell compression in growth control (i.e. the transition from a growing cell to a quiescent cell). While cell volumes do not necessarily change, resolving these compressive forces is crucial to correctly model tumour spheroid growth."

***Response to the minor comment-4

W appreciate the reviewer's comment. First, the choice of the term "compressive" could have been misleading. We had meant significant reduction of cell volume by "compressive". But, as far as we have carefully checked again our simulation data, we cannot obtain solid evidence that cell volumes are indeed reduced. Therefore, we have retracted the descriptions about the cell volume reduction.

Alternatively, we have found that cell positions are fixed and show a well-aligned array throughout the simulations in the cases of the lengths of the spherocylinders = 16 and 20μm (Fig. 3-i in the revised manuscript, broken lines), resembling a closed-packed state. Although tissues under compressive conditions exist such as spheroids, we do not know the existence of tissues where cell movements are absolutely restricted by external spatial constraints. We have mentioned above in our revised manuscript.

Line 290; "In Fig. 3-i, the particle diameters were depicted to be equivalent to the mean

diameter of cells, and, under the condition of the length = 16μm, the distances between the adjacent particles seemed to be less than the diameter of the particles, meaning a compressed state. Moreover, the positions of the particles were not changed but fixed throughout the simulations (Fig. 3-i, 16μm), and showed a well-aligned array resembling a closed-packed state (Fig. 3-i, broken lines). Compressive states are observed in real tissues such as spheroids [29,36,37], whereas we do not know the existence of tissues where cell movements are absolutely restricted by spatial constraints; if such tissues existed, DF curves could be affected by the constraints."

*****Minor comment-5: "Line 291: What does it mean to essentially have a unique solution?"

***Response to the minor comment-5

Thank you for your comment. In general, when we perform a fitting problem, we should consider whether the problem has a unique solution but not multiple solutions. In our case, we obtained a set of force values by fitting, which can reproduce the in vivo xyz-coordinates. But, if there could be other sets of force values, which can also reproduce the xyz-coordinates, different trials of our fitting procedure would result in different solutions by chance. Such indefiniteness is not desirable, because we cannot judge which solution is plausible. In the field of fitting problems, researchers consider this issue as previously discussed (1. Bayesian inference of force dynamics during morphogenesis, Ishihara & Sugimura, JTB, 2012, 2. Video force microscopy reveals the mechanics of ventral furrow invagination in Drosophila., Brodland et al., PNAS, 2010). In our manuscript, we had performed multiple trials of our fitting procedure under different initial conditions, and found that the solutions were equivalent each other, suggesting the uniqueness of solutions (Fig. 4D and S5 in our original manuscript). In our revised manuscript, we have mentioned about the importance of the uniqueness with the above references.

Line 318; "Note that, in our inference in this system, we confirmed that this inference problem has essentially a unique solution (Fig. 4D); the uniqueness of solutions is important to judge whether the inferred result is plausible in fitting problems as previously discussed [39,40]."

*****Minor comment-6: "Line 457: There are also 3D vertex models see the work by Durney and Feng."

***Response to the minor comment-6

Thank you for introducing a paper. We have added it in our revised manuscript.

> Line 539; "Epithelial cells are usually modeled by the vertex models where each cell is assumed to be polygonal, and many-body (i.e. ≥3 cells) interactions but not pairwise ones are considered [62,63]."

*****Minor comment-7: "Line 65: parameters at the cellular level"

***Response to the minor comment-7
Thank you for your suggestion. We have revised our manuscript.

*****Minor comment-8: "Line 70: interactions are considered."

***Response to the minor comment-8
Thank you for your suggestion. We have revised our manuscript.

*****Minor comment-9: "Line 141: we develop a top-down method"

***Response to the minor comment-9
Thank you for your suggestion. We have revised our manuscript.

*****Minor comment-10: "Line 150 and following: On first look cells in embryos appear highly changing (in shape, position etc), maybe the authors could add a sentence to clarify the differences between epithelial, mesenchymal and blastomeres and their properties here."

***Response to the minor comment-10
We appreciate the reviewer's suggestion. As the reviewer pointed out, deformability of cells would matter. Epithelial and mesenchymal cells often show highly deformed shapes (i.e., far from spherical/circular shapes). This is because vertex models, which can explicitly present the highly deformed shapes, are favored for modeling epithelial cells, instead of particle models. By contrast, blastomeres tend to show nearly spherical shapes. Therefore, we had expected that fitting of particle models could work for blastomeres. We have explained the above differences in our revised manuscript.

Line 159; “We assume that the nearly-spherical cell shape of blastomeres may be well-approximated by particle models, while the highly deformed cell shapes of epithelial or mesenchymal cells may not be suitable. This issue will be discussed later in the Discussion section.”

In addition, in systems where external forces (e.g., traction forces exerted between cells and ECM) substantially contribute to cell movements, inferences of cell–cell interaction forces would be affected, as mentioned in the discussion section of our original manuscript.

*****Minor comment-11: “Line 164: which originate from”

***Response to the minor comment-11
Thank you for your suggestion. We have revised our manuscript.

Reviewer #2

****

Comments about “Writing”

****

*****Comment-1: “Line 108: “However, mechanical parameters … remain …”

***Reponses to the comment-1

Thank you for the comment. We have revised our manuscript.

*****Comment-2: “Rephrase Lines 113-121: The end of the first sentence “as follows” suggests a list of actions. The next sentence breaks the flow. A potential following could be “Cell-cell adhesion forces, which are …””

***Reponses to the comment-2

As suggested by the reviewer, we have revised our manuscript; we have deleted the “next sentence” that the reviewer pointed out.

*****Comment-3: “Line 118: Clarify “whereas both excluded volume effect …””

***Reponses to the comment-3

As suggested by the reviewer, we have explained as follows.

> Line 123; “due to cell volume conservation, two cells cannot extremely approach each other, and the cell surface tensions can antagonize cell–cell adhesion forces.”

*****Comment-4: “Line 122: Did you mean “pairwise potentials of objects such as …””

***Reponses to the comment-4

Yes, thank you for the comment. We have revised our manuscript.

*****Comment-5: “Figure 4F,G: Did you mean “cell-cell distance”?”

***Reponses to the comment-5

Yes, thank you for the comment. These are our mistakes, and we have revised our manuscript.

*****Comment-6: “Lines 301-303: Are the potentials normalized by distances? If not, please rephrase.”

***Reponses to the comment-6

Yes, we had normalized the potentials by distances: the unit of potential energy contains meter^2, and thus, one can normalize the component of meter^2 in the potential energy by meter. We have added this explanation in our revised manuscript.

> Line 333; “the potentials were divided by the square of the distances at the potential minima.”

*****Comment-7: “Line 426: “Our findings provide for the first time …”. Kindly consider https://doi.org/10.1101/2022.11.05.515310”

***Reponses to the comment-7

We appreciate the reviewer’s comment. The reviewer mentioned about an article entitled “A mathematical theory for the mechanics of three-dimensional cellular aggregates reveals the mechanical atlas for Ascidian embryogenesis. Liu et al., bioRxiv”. Liu et al. has developed an image-based force inference method in ascidian embryos, where line tensions of cell–cell interface etc. are inferred. At line 426 in our original manuscript, because we had discussed about inference of pairwise potential but not line tensions, citing the Liu’s paper at line 426 is not appropriate. Alternatively, we have cited the Liu’s paper at a different position as follows.

Line 533; “Possibilities of image-based inference methods are expanding under three-dimensional situations [61].”

*****Comment-8: “Line 458-459: “It remains unknown whether pairwise potentials can be …”. Kindly consider

https://doi.org/10.1371/journal.pone.0254763,

https://doi.org/10.1016/j.cels.2019.02.007”

***Reponses to the comment-8

Thank you for suggesting interesting papers.

The first paper entitled “Computational modelling unveils how epiblast remodelling and positioning rely on trophectoderm morphogenesis during mouse implantation” presented a model in which a single cell was described by multiple particles (~30) but not a single particle. Therefore, although interactions between the particles were defined by potentials, this is different from the potential of pairwise cell–cell interactions. This model is on a similar line of the Kajita’s paper describing a single cell by a network of a hundred of particles (ref. 47 in our original manuscript). Therefore, we have cited the paper in the section of the comparison of other models in our original manuscript.

The second paper entitled “ya||a: GPU-Powered Spheroid Models for Mesenchyme and Epithelium” proposed a simulation software based on a particle model. A pairwise potential of cell–cell interactions was also implemented, and simulations of large tissues were demonstrated. In our manuscript, the advantage of particle-based model for simulating large tissues is discussing, and so, we have cited the paper in our revised manuscript.

> Line 518; “These models successfully recapitulated various morphogenetic events, including cell sorting, epithelial cell movements/rearrangements, the formation of the mouse preimplantation embryos [53,57] ,…”

> Line 543; “…though three-dimensional epithelial cells were modeled by particle-based models, which enabled one to simulate three-dimensionally large tissues [15,18,19,64].”

*****Comment-9: “Line 467-468: Did you mean “It is important … factors affect inferenced”

***Reponses to the comment-9

We have revised our manuscript as follows; “…factors affect inferred results…”

****

Comments about “Method”

****

*****Comment-10: “Lines 144-145: “Our method was validated by using various synthetic data generated by simulations assuming cell proliferation”. How was this synthetic data simulated? Why not validate with a subset of the real dataset?”

***Reponses to the comment-10

Thank you for the reviewer's comment. We think that the sentence may be a little bit obscure. First, there seem to be a few discrepancies. We did not mention "the synthetic data was simulated.". Rather, we mentioned that, by simulating particle models, we generated the synthetic data. The simulation procedures had been provided in the supplementary information of our original manuscript. Importantly, in these simulations, we had set the Lenard-Jones potential as the cell–cell interaction potentials. Therefore, we know the correct potential in the systems, and the inferred potential should become equivalent to the correct potential. This feature is critical to judge whether our method can infer the correct potentials of systems. By using the above idealized conditions, we had validated our method. By contrast, in real dataset (i.e., in vivo data), we do not know the correct potentials, because there is no other method for obtaining the potentials in multicellular systems. Under these situations, it is difficult to validate our method. We have revised our manuscript as follows.

> Line 151; "Our method was validated by using various synthetic data which were generated by simulations under pregiven cell–cell interaction forces ."

*****Comment-11: "How would you adapt the model and the cost function for non-constant physical conditions (e.g. ¥lambda variable)"

***Reponses to the comment-11

We believe that the reviewer is referring to cases where "gamma" value but not "lambda" is non-constant, because we did not use lambda in our manuscript. Indeed, we agree that gamma could be non-constant in real tissues. However, unfortunately, it is challenging to measure spatiotemporal distributions of the gamma values, as previously reported (ref. Spatial mapping of tissue properties in vivo reveals a 3D stiffness gradient in the mouse limb bud., Zhu et al. PNAS, 2020). Therefore, in our manuscript, we had assumed the simplest situation; gamma = const. (=1.0), as described in our original manuscript. This is a common problem in image-based force inference methods to be solved in future (ref. 1. Bayesian inference of force dynamics during morphogenesis, Ishihara & Sugimura, JTB, 2012, 2. Video force microscopy reveals the mechanics of ventral furrow invagination in Drosophila., Brodland et al., PNAS, 2010), except for the cases where quasi-static processes are assumed. In our revised manuscript, we have additionally mentioned that gamma can be spatiotemporally non-constant in real tissues.

> Line 185; "In addition, gamma may be not constant in real tissues, while it is challenging to measure the value with spatial resolution at cellular level [30]."

> Line 554; "In our inference method, the coefficient of viscous frictional forces is considered as described in Equation 1. Typically, this coefficient is assumed to be constant in

multicellular models. However, the values of this coefficient and its spatiotemporal distributions in real tissues remain poorly understood [30]. There can be many factors affecting the coefficient; e.g., cell–cell contact area, cell size, the number of contacting cells per one cell, cell–cell adhesion molecules including cadherins, surface structures of cells [65–67]. In the second model presented in the C. elegans embryos (Fig. S8), the number of contacting cells per one cell is effectively considered. Influence of cell–cell adhesion molecules means that the coefficient is different among cell types (e.g., differentiated vs. undifferentiated cells, etc.). Unfortunately, experimental measurements of the coefficient with spatiotemporal resolution are lacking."

*****Comment-12: "How does the method behave with simulated data obtained from different potentials (e.g. in addition to LJ, Morse potential, …)? Other than the visual appreciation of the resemblances between real and reconstructed DF curves, is there a quantitative metric (e.g. L2 norm)?"

***Reponses to the comment-12

We agree with the reviewer's comment. In our original manuscript, we had tested the case of different potentials in addition to LJ (Fig. S2 which was mentioned in the legend of Fig. 2). We have further tested the Morse potentials in our revised manuscript, and found that the inferred potentials were similar to the Morse potential.

As suggested by the reviewer's, we have calculated the L2 norm to evaluated the differences of the inferred DF curves and the LJ potential.

Line 252; "We also quantitatively evaluated the differences of the force values between the inferred DF curves and the LJ potential by calculating L2 norm which is described in the legend; i.e., L2 norm = $\{F_{\mathrm{inferred}}(D) - F_{\mathrm{LJ}}(D)\}^2$, where F_{inferred} and F_{LJ} are the force values from the inferred DF curves and the LJ potential, respectively."

Line 257; "In addition to the LJ potential, we also tested other potentials (e.g., Morse potential) and the inferred DF curves were similar to the given potentials (Fig. S2 and S3)."

*****Comment-13: "Two cases were identified for which the method did not produce thrilling results: longer time intervals, and restricted space. Do these observations have any bearings on the overall validity of the method?"

***Reponses to the comment-13

We appreciate the reviewer's comment. As pointed out by the reviewer, under the conditions of longer time intervals and highly restricted space, our inference became to show larger discrepancies to the given potential. These results just show the limitations of our method. In general, any methods have a lot of limitations, and users should apply the method within the permissive conditions. For instance, mathematical models must be simulated under very shorter time intervals, because longer time intervals result in incorrect simulation outcomes. Force estimation by using laser ablation experiments should be also performed within shorter time period after the ablation, because active cellular processes become non-negligible for longer time period. In other words, there is no method which is applicable for any conditions. We believe that describing limitations are very important which should not be hidden, and such information is positive but not negative for method papers.

*****Comment-14: "How is division handled?"

***Reponses to the comment-14

As pointed out by the reviewer, we had developed a framework for handling cell division. In addition, handling dead/lost cell is also required. In our original manuscript, we had described the related procedures in the supplementary information. Because the procedures are too complicated to explain in the main text, we have cited the supplementary information in our revised manuscript.

> Line 266; "…handling of cell proliferation/division is described in the Supplementary information with the case of dead/lost cells."

****

Comments about "Application in C. elegans and mouse"

****

*****Comment-15: "Fig 4G: Could you comment on the slight deviations observed for stages t36-75 and t76-115. Are these deviations significant enough to infer different mechanical conditions in the embryo during these stages?"

***Reponses to the comment-15

Thank you for the comment. But, at this moment, we do not know whether the slight deviations are meaningful or not. Also, we do not know whether there is a mechanical change among the embryonic

stages. We have added a following sentence in our revised manuscript.

> Line 336; "Although slight deviations were observed for the stages t36-75 and t76-115, whether this represents changes in the mechanical properties in the embryo requires further investigation."

Reviewer #3

*****Comment-1: "An important limitation is that forces are considered as pair-wise potentials, this means that for a given contact pair, the force is only dependent on state variables of the two interacting particles, but not the surrounding. In reality, this assumption will break down for the interaction between highly deformable bodies, in which the mechanical state is determined by the balance of surface tension and adhesion across all contacts. For example, a doublet that is laterally confined by other cells will not adhere to the same extent as an isolated single cells. For stiff particles / colloidal particles, this limitation does not play a role, since deformations are strongly localized (e.g. Hertz assumptions) and the pair-wise potential still holds. However, this is not expected for many cell types, certainly those that are typically modeled using vertex-like models. I think the authors should investigate the limitations of their approach for these highly deformable cells (preferably quantitatively) and discuss these in the manuscript."

***Reponses to the comment-1
We agree with the reviewer's comment that our approach may not be suitable for highly deformable cells, and that the vertex models have used for such highly deformable epithelial cells.

We have investigated the applicability of our method to highly deformable cells; our inference method has been applied to the synthetic data generated by simulating a vertex model (Fig. R1 below for review). Surprisingly, even in the cells of the vertex model, distance–force curves were clearly detected (Fig. R1B-ii). Moreover, cell–cell adhesion forces in the vertex model showed a positive relationship with the attractive forces in the distance–force curves (Fig. R1B-iii), which includes a quantitative evaluation. By contrast, line tensions between cell–cell junctions in the vertex model showed a negative relationship with the attractive forces (Fig. R1C-ii). These relationships are basically the same as those in isolated two cell systems (Fig. 1 in our manuscript), indicating that the particle model can approximate the highly deformable cells to some extent.

In spite of the above results, we assume that there may be phenomena which are reproduced by vertex models but not so well by particle models, which could include fluidity of tissues, behaviors about jamming, cell–cell junctional rearrangement, etc. (ref. Motility-driven glass and jamming transitions in biological tissues. Bi et al. PRX, 2016). We think that the above topics contains too much information to merge into our present manuscript, and is beyond the scope of our present manuscript. In our original manuscript, although we had mentioned in the discussion section, that "it remains unknown whether pairwise potentials can be a good approximation of epithelial cells…", we had not clearly discussed about the high deformability. We have thus referred to this issue and clearly stated the potential limitations of our approach in the revised manuscript.

Line 159; "We assume that the nearly-spherical cell shape of blastomeres may be well-

approximated by particle models, while the highly deformed cell shapes of epithelial or mesenchymal cells may not be suitable...”

Line 541; “By using the vertex model, the highly deformable features of epithelial cells are well reproduced.”

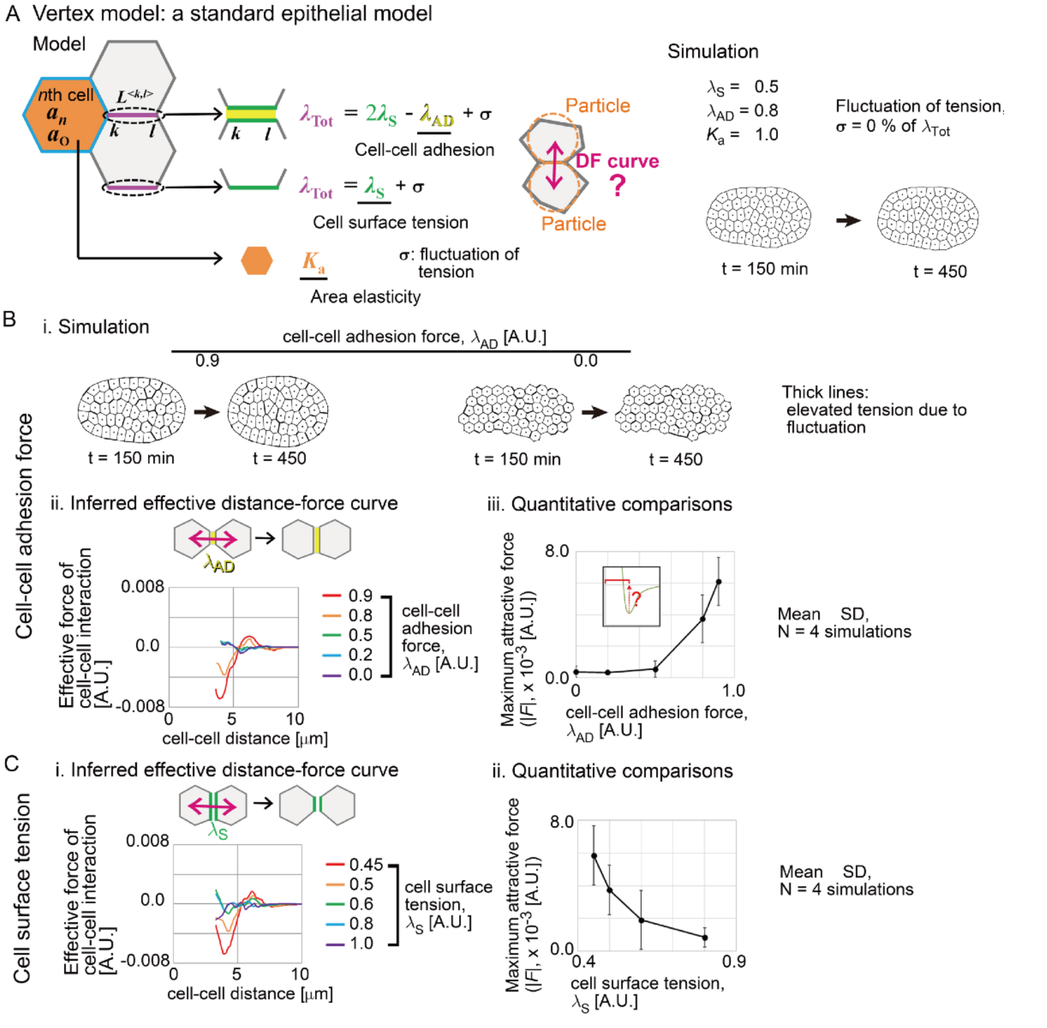

Fig.R1 Koyama et al.

Figure R1: Inference of effective forces of cell–cell interaction in vertex model

A. Vertex model. λ_{Tot} is the line tension exerted on cell–cell junctions or on cell edges of outer boundary. The potential energy on a junction or edge is $\lambda_{Tot} L^{<k,l>}$, where L is the length of the junction or edge composed of kth and lth vertices. The potential energy for a cell provided by the area elasticity is $(1/2)K_a\left(a_n/a_o-1\right)^2 a_o$, where an is the area of nth cell and a_o is the natural area. An example of simulations is shown in the right panel.

B. Inferred DF curves under various values of cell–cell adhesive forces, λ_{AD}. i) Simulation outcomes are shown where the cell cluster tends to form a circular colony under increased values of the adhesive forces ($\lambda_{AD} = 0.9$). ii) Inferred DF curves. iii) Quantitative comparisons of the DF curves.

C. Inferred DF curves under various values of cell surface tensions, λ_S. i) Inferred DF curves. ii) Quantitative comparisons of the DF curves.

*****Comment-2: “In the same line of reasoning, their model assumes there are no contact-specific state variables (i.e. contact state) that play a role since the potential only takes into account the positions of the particles. In reality, contacts between cells involve bonds, which give rise to a clear hysteresis upon approach / retraction. This hysteresis is for relatively stiff particles captured in JKR-like potentials and for liquid-droplet / foam-type models will play an even bigger role. The authors should motivate their choice of neglecting this hysteresis. Effectively, it implies that the cell is always able to 'probe' its neighbourhood and react mechanically in accordance, even at a distance away from the cell (determined by the shape of the potential)”

***Reponses to the comment-2

Thank you for the interesting point. We absolutely agree that cell–cell interactions would show hysteresis. Due to hysteresis, cell–cell interaction forces can be different even under the same cell–cell distance. For instance, when two adhered cells are dissociating each other, the attractive force can remain around longer cell–cell distance where cell shapes may be highly elongated toward the cell–cell contact site. By contrast, when two separated cells are approaching each other, the attractive force caused by cell–cell adhesion becomes exerted around relatively shorter cell–cell distance. Therefore, distance–force curves for the above two cases become different. Actually, this behavior is observed when measuring cell–cell contact forces by the atomic force microscopy etc.

It seems that the reviewer felt that we neglected the hysteresis, but this is not the case. Our method temporally inferred cell–cell interaction forces; i.e., at each time frame. Therefore, we can distinguish whether each cell–cell interaction is approaching or dissociating, and then, we can, in principle, investigate whether there is hysteresis in the systems. In our present manuscript, we had presented a “mean” distance–force (DF) curve; i.e., averaging two hidden DF curves from approaching and dissociating two cells. This is because the morphologies of the systems in our manuscript were reproduced by the mean DF curves.

As described above, our method contains temporal information on cell–cell interaction forces, and could be useful for studying systems with hysteresis and its possible contribution to phenomena such as jamming. We thus agree with the reviewer that the issue of hysteresis is of potential interest and is an exciting area to explore the utility of our method in the future. On the other hand, we consider that this topic is beyond the scope of the present study. Thus, in our revised manuscript, we discuss this issue in depth to provide perspectives to the readers.

Line 545; “In deformable objects such as cells, cell–cell interaction forces show hysteresis; i.e., the forces at the same distance become different between the cases when two cells are approaching and dissociating. This effect may be expressed by different profiles of DF curves between the two cases. In the vertex models, this hysteresis is implemented through its high deformability. Because our inference method contains temporal information of

cell–cell interaction forces, it may be possible to examine whether systems show hysteresis. It would be worth investigating whether the framework of two DF curves based on the hysteresis can expand the capability of particle models for describing various phenomena."

*****Comment-3: "Another important limitation in the potential is the lack of a velocity-dependent component in the interaction force. Typically, individual cell-based models take these velocity-dependent forces into account, as relative frictional forces, see e.g. this old work:

Drasdo, Dirk, Stefan Hoehme, and Michael Block. "On the role of physics in the growth and pattern formation of multi-cellular systems: what can we learn from individual-cell based models?" Journal of Statistical Physics 128 (2007): 287-345.

In case of C. elegans, it makes sense that there are strong viscous contributions that determine the relative velocities (and thus relative displacements) of adjacent cells. For example, the cortical material of the egg has revealed a hydrodynamic length-scale of 68 um.

Saha, Arnab, et al. "Determining physical properties of the cell cortex." Biophysical journal 110.6 (2016): 1421-1429.

If this is the case, viscous forces can be propagated over long distances in the embryo and the intercellular potential will not only be determined by the relative distance between two cells, but also by their relative velocity. It would greatly benefit the generality of this paper if the authors assess the effect of such a contribution in their model. In this case, Eq. 1 would not be correct, as this equation uses a single global damping coefficient gamma (representing some kind of medium damping), rather than a viscosity that would be dependent on the relative velocity of the two cells. For C. Elegans, these relative velocities are available, so it would be a matter of including this additional parameter in their model."

***Reponses to the comment-3
Thank you for raising this issue. The reviewer pointed out that viscous frictional force should be determined by the velocity of an object relative to its surrounding environment but not by the absolute velocity of the object. For instance, given that the origin for generating the viscosity is the surrounding objects, if the object of interest and the surrounding objects move with the same velocity, the viscous frictional force exerted on the object of interest should become 0. On the other hand, if the surrounding environment generating the viscous frictional forces is non-moving liquid medium (i.e., its velocity =

0), the forces are described by the absolute velocity of the object of interest (i.e., in this case, the relative velocity becomes the same as the absolute velocity). We agree with this concept.

As pointed out by the reviewer, the relative velocity model is adopted in some papers. On the other hand, the absolute velocity model is also adopted in other papers (ref. 18-20 in the revised manuscript). In the case of the vertex models, the absolute velocity model is dominantly adopted, but the relative velocity model is also presented (ref. Vertex dynamics simulations of viscosity-dependent deformation during tissue morphogenesis. Okuda et al. Biomech Model Mechanobiol, 2015). Although the viscous friction is a well-known concept in multicellular modeling, we think that the origin of the friction is controversial due to the lack of experimental determinations. Therefore, the experimental values of the viscous friction coefficient (gamma) are unavailable for both particle models and vertex models.

As suggested by the reviewer, to expand the generality of our manuscript, we have developed an inference method based on the relative velocity model in our revised manuscript. In this new method, we have designed so that its cost function to be minimized is the same as that adopted in the original method based on the absolute velocity model. We have applied this new method to the C. elegans embryos, and showed that the inferred distance–potential curves are similar to those inferred by the original method (Fig. S8A in the revised manuscript). We think that this is because collective migration of cells (i.e., systems with similar velocities among the cells) rarely occur during the early developmental stages in C. elegans. We speculate that, in systems of collective migration, inferred DF curves may become significantly different.

Note that, in our new method, the absolute value of the viscous friction coefficient (gamma) has become to affect the profile of the inferred DF curves (Fig. S8B), whereas, in the original method, the absolute value did not affect. Unfortunately, we cannot find a reasonable logic to uniquely determine the absolute value, but, the profiles of the inferred DF curves showed almost similar each other within the range of 10^3 of gamma (Fig. S8B in the revised manuscript). In the revised manuscript, we have shown the above data with discussions.

Fig. S8

Line 349; "Model dependency of inference in C. elegans embryo. Next, we evaluated a model-dependency. In our particle model, the viscous frictional forces were assumed to be proportional to the absolute velocities of the particles as previously presented (Eq. 1). On the other hand, some papers argued that the viscous frictional forces should be determined by the velocities relative to surrounding particles [29,36]. The equation of motions in this model is as follows; $F_{C|p} = \sum_{m=1}^{N} [\{\omega_g(D_{pm})\gamma\}\{(V_{C|p} - V_{C|m})\bullet e_{pm}\}e_{pm}], \ldots$"

Line 554; "In our inference method, the coefficient of viscous frictional forces is considered as described in Equation 1. Typically, this coefficient is assumed to be constant in

multicellular models. However, the values of this coefficient and its spatiotemporal distributions in real tissues remain poorly understood [30]… In the second model presented in the C. elegans embryos (Fig. S8),…"

*****Comment-4: "On line 210 it is explained that the cost function Eq. 3 'worked well'. The authors should in the main text specify more clearly what it means to 'work well', and why the previous proposal does not 'work well'."

***Reponses to the comment-4

As suggested by the reviewer, we have revised our manuscript as follows.

Line 220; "Equation 2 did not work well: the inferred forces were not consistent with given potentials. In particular, even around the longer cell–cell distance, the force values did not decay (Fig. S2)."

*****Comment-5: "Small remark, but the shape in Fig. 3 is a spherocylinder or capsule, not a cylinder."

***Reponses to the comment-5

Thank you for your suggestion. We have revised our manuscript according to the suggestion.

*****Comment-6: "I do not agree with the reasoning in line 266-270. There are cells that can live in a compressed context and it is not because the cytosol itself is incompressible that cells cannot be in a general compressed state (meaning that they exert a net repulsive force on their neighhourhood). More weakly, it is true that in most tissues, a tensile state is observed (if the boundary conditions permit). However, in some cases it is obvious that local compressed states must exist: For example, take a free tissue spheroid or cell aggregate: The cells at the periphery will generate a net tissue surface tension. Young-laplace law dictates that the core of the tissue spheroid must be under pressure, and that, on average, these cells must exist in a compressed state, see e.g.

Cuvelier, M., Pešek, J., Papantoniou, I., Ramon, H., & Smeets, B. (2021). Distribution and propagation of mechanical stress in simulated structurally heterogeneous tissue spheroids. Soft Matter, 17(27),

6603-6615.

I think this reasoning should be removed from the paper as it does not further benefit the presentation of the results."

***Reponses to the comment-6

Thank you for the reviewer's comment. We agree with the comment. First, the term "compressive" may not have been accurate in our original manuscript. We had meant significant reduction of cell volume by "compressive", while we know that, in studies of spheroids, compressive conditions are considered where we guess that significant reduction of cell volume does not occur.

In response to the reviewer's comment, we have carefully checked again our simulation data under the compressive conditions. Unexpectedly, we cannot obtain solid evidence that cell volumes are indeed reduced. Instead, we have found that cell positions are fixed and show a well-aligned array throughout the simulations in the cases of the lengths of the spherocylinders = 16 and 20μm (Fig. R2 below, broken lines), resembling a closed-packed state. We know tissues under compressive conditions, whereas we do not know the existence of tissues where cell movements are absolutely restricted by external spatial constraints. We have mentioned above in our revised manuscript.

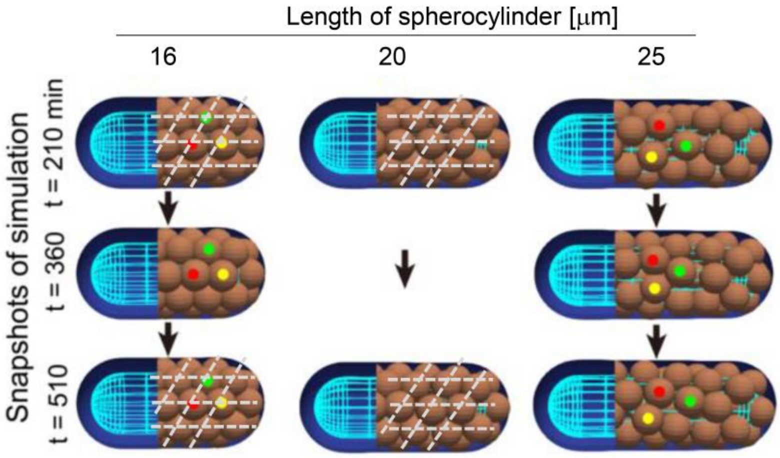

Figure R2: Movement of particles in confined conditions
Snapshots of simulations in Fig. 3 are shown. In the case of the length of spherocylinder = 25μm, the positions of the particles are changing during the simulation. By contrast, in the cases of the length = 16 and 20μm, the positions are fixed. Three particles are labeled in the case of 16 and 25μm (ref, green, and yellow). The particles in the cases of 16 and 20μm show ordered arrays as marked by the broken gray lines, and these arrays are maintained during the simulations.

Line 293; "Moreover, the positions of the particles were not changed but fixed throughout the simulations (Fig. 3-i, 16μm), and showed a well-aligned array resembling a closed-

packed state (Fig. 3-i, broken lines). Compressive states are observed in real tissues such as spheroids [29,36,37], whereas we do not know the existence of tissues where cell movements are absolutely restricted by spatial constraints; if such tissues existed, DF curves could be affected by the constraints."

*****Comment-7: "I am not convinced by the results presented in Fig 6 and Fig 8, which aim to show that for the measured differences in cell potential, different behaviors of cell assemblies will be expected. I think these figures are very qualitative and miss any depth or physical interpretation. If the aim is truly to investigate the difference between these potentials on tissue morphology, the rheological and physical properties of these tissues arising from the difference in potential should be investigated. E.g. the difference in tissue surface tension, shear rigidity, apparent elasticity. Also, it is expected that glassy states emerge for such sphere-approximations of cells. In practice these could be overcome by actual deformable cells and actual cell activity, which is not taken into account in this. I suggest that the authors remove these figures to the supplementary information, as they only make a weak point on the effect of these differences in potential."

***Reponses to the comment-7
Thank you for the reviewer's comment. As pointed out by the reviewer, we had not analyzed tissue surface tension etc. We agree that the relationship of the pairwise potential with the rheological and physical properties of tissues can be of great interest especially in physics, and so we want to analyze it. But in this paper, we are mainly focusing on the morphologies of tissues but not the rheology, because we think that the link of mechanical parameters with tissue morphologies is important especially in biology.

Tissue surface tension contributes to the rounding-up of tissues (ref. 1. Rounding of aggregates of biological cells: Experiments and simulations. Mombach et al., Physica A, 2005; 2. Differential adhesion in morphogenesis: a modern view. Steinberg. Curr. Opin. Gen. & Dev. 2007; 3. Measuring accurately liquid and tissue surface tension with a compression plate tensiometer. Mgharbel et al. HFSP J. 2009. etc.); in analogy to liquid droplets. According to the theory of surface tension in liquid droplets and soap bubbles, even under slight surface tensions, the objects will finally round up. Basically, the previous works revealed that, when non-spherical cell aggregates were given, the aggregates tended to round up to spherical shapes, where the speed of the rounding-up was correlated with tissue surface tension (i.e., dynamics of rounding-up). Although the presence of tissue surface tension may be a generic property of tissues as previously mentioned (ref. 1. Dissipative particle dynamics simulations for biological tissues: rheology and competition. Basan et al. Phys. Biol. 2011), tissues do not always exhibit spherical shapes, including the C. elegans embryos whose eggshells are

removed (ref. 44 and 45 in the main text). Our manuscript had also showed that the drugs-treated mouse embryos presented a morphological transition from a spherical shape to a non-spherical shape after adding the drugs (Fig. S11), indicating that the drugs-treated embryos preferred a non-spherical shape. Therefore, there are other factors which can significantly overcome the effect of the tensions. On the other hand, according to excellent papers demonstrating what the origins of tissue surface tension are (ref. 1. Coaction of intercellular adhesion and cortical tension specifies tissue surface tension. Manning et al. PNAS, 2010; 2. Quantitative differences in tissue surface tension influence zebrafish germ layer positioning. Schötz et al. HSFPJ. 2008), tissue surface tension becomes higher by increased cell–cell adhesion forces. Because the pairwise potentials of cell–cell interactions should include the effect of cell–cell adhesion forces, our analyses may contain the effect of tissue surface tension.

In addition, the EDTA-treated mouse embryos harbor intercellular spaces (Fig. 7D), in which we suppose that tissue surface tension is not correctly defined; please imagine an extreme case that, in an assembly of magnetic metal beads, can surface tension be correctly defined? In our manuscript, we presented a concept for tissue rounding in tissues with intercellular spaces. We fortunately found the direct link of the profiles of pairwise potentials to sphericity of tissues. Also, we believe that the related figure (Fig. 8B) quantitatively shows a very clear relationship; from Fig. 8B, we can read the control parameter, the order parameter, and the critical point.

Finally, although we agree that shear rigidity and apparent elasticity are important parameters for tissue rheology and physical properties, we do not know any previous reports or theories where shapes of objects are determined by these parameters. For instance, elasticity of objects does not determine the shape of the objects (e.g., spring). Therefore, we cannot expect to draw a parameter space showing the relationship between shapes and the parameters such as elasticity. Similarly, we cannot find any data from previous reports, showing a parameter space between tissue surface tension and shapes.

Line 494; "Morphology of embryo. For the generation of spherical/circular tissues, tissue surface tension is involved in [1,29,49,50]. The surface tension reduces surface area of tissues in analogy to liquid droplets. Therefore, when non-spherical cell aggregates are provided, they show a tendency to round up to spherical shapes, where tissue surface tension contributes to the dynamics (i.e., the speed of the rounding-up) [49,51]. Although the presence of tissue surface tension may be a typical property of tissues [1,29], tissues do not always exhibit spherical shapes including the C. elegans embryos whose eggshells are removed [44,45]. Similarly, the drugs-treated mouse embryos showed a morphological transition from a spherical shape to a non-spherical shape after adding the drugs (Fig. S11). These data mean that tissue surface tension is not a sole determinant, but there are other factors significantly contributing to tissue shapes which can overcome the effect of the

tensions. Note that cell–cell adhesion forces contribute to both tissue surface tension [26,50] and the pairwise potential of cell–cell interactions (Fig.1), suggesting that the pairwise potential relates to tissue surface tension.

In addition, the EDTA-treated mouse embryos harbor intercellular spaces (Fig. 7D and S13), in which we do not think that tissue surface tensions are correctly defined. Our result identified a parameter which explains sphericity of such tissues (Fig. 8B). Similar tissues with intercellular spaces are also known in other species and tissues including zebrafishes [52]."

*****Comment-8: "The derived potentials (e.g. fig 4) look very interesting. However, it is a shame that the authors did not quantify the properties of these potential in more detail and do some comparison with classical models used in cell-based simulation. For example, it looks like these potentials more closely correspond to a model such as used in:

Delile, J., Herrmann, M., Peyriéras, N., & Doursat, R. (2017). A cell-based computational model of early embryogenesis coupling mechanical behaviour and gene regulation. Nature communications, 8(1), 13929.

(see supplementary figure 4 for that paper, invert the y-axis), which is commonly used in cell-based simulations these days, and it looks less similar to e.g. JKR or similar model descriptions.
Could the authors do an attempt to fit these very simple mathematical models to their derived potential and discuss eventual discrepancies?"

***Reponses to the comment-8
We appreciate the review's comment. As pointed out by the reviewer, evaluation of differences among our inferred potentials and previously-used potentials is of much interest. In the paper which the reviewer raised (Delile et al.), Delile et al. proposed a new framework of distance–force curves. In their supplementary figure 4, their framework was shown with a comparison of the Morse potential. We think that their framework is not commonly used but the Morse potential is often used (ref. 13,18,20 in the revised manuscript). Because the Morse potential is a quite flexible framework by which distance–force curves with various profiles can be generated. For instance, the Morse potential used in Nissen et al. (ref. 18,19 in our revised manuscript) shows a very long range of cell–cell distances where attractive forces are exerted, whereas the Morse potential presented in Delile et al. shows a relatively short range though it still shows a longer range compared with the Lenard-Jones potential (Fig. S3 in our revised manuscript). Note that, as long as we have tried, the Morse potential

can be fitted well to the Lenard-Jones potential, due to its flexibility (data not shown).

In response to the reviewer's comment, we have fitted the above two framework (i.e., the Delile's framework and the Morse potential) to our inferred potentials. Basically, the Morse potential was fitted to our inferred potentials with slight discrepancies (Fig. S7 in our revised manuscript). The resultant curves have shown a relatively short range of distances where attractive forces are exerted, compared with the Delile's and Nissen's ones (Fig. S3C, D vs. Fig. S7A in our revised manuscript). On the other hand, the Delile's new framework have not been well fitted to our inferred potentials (Fig. S7B). This is because the Delile's potential sets a narrower range of cell–cell distances where attractive forces can be exerted. We have mentioned about the above analyses in our revised manuscript.

Line 339; "We also compared the profiles of the inferred DP curves with previously used ones. The framework of the Morse potential is often considered (e.g. Fig. S3) [13,18,20], while other frameworks were presented [14,16,20,29,41]."

Fig. S3C and D; the Delile's and Nissen's Morse potentials are shown.

Fig. S7; the Morse potential or the Delile's framework was fitted to our inferred potentials (A or B, respectively).

In addition, as pointed out by the reviewer, quantitative differences of the profiles of our inferred potentials are of interest. As shown in our original manuscript (Fig. S11), we had quantitatively quantified the profiles. Moreover, although we had not shown in our original manuscript, we have other quantitative data: decay of attractive forces, stiffness of particle's core. We have values of these indices in the C. elegans, mouse embryos with or without the drugs-treatment, the LJ, and the FH (used in Fig. S2) potentials. In our manuscript, we had chosen the indices to be presented, which showed a relationship to the shapes of tissues, because too many indices would comprise the readability of our manuscript. In future, we want to present comprehensive analyses about the indices in various tissues.

Finally, we really appreciate the reviewer's insightful comments from the viewpoints of various aspects; deformability of epithelial cells, hysteresis, modeling viscosity, rheology, compressed tissues such as spheroids, quantification of the profiles of the potentials. We agree that all of them are important issues. But we feel that the presentation of all these data into our present manuscript will make the focus of our manuscript vague. We want to study the above topics in future.
