# Supplementary material for: Effective mechanical potential of cell–cell interaction explains three-dimensional morphologies during early embryogenesis
Source: PLoS Comput Biol. 2023 Aug 7;19(8):e1011306. doi: 10.1371/journal.pcbi.1011306 (PMC10434874; doi:10.1371/journal.pcbi.1011306)
Supplement: Attachment — Submitted filename: Paper_Review.docx [file pcbi.1011306.s023.docx]

Review: Effective mechanical potential of cell-cell interaction explains three-dimensional morphologies during early embryogenesis

The paper addresses an important question in the field which is how to quantify mechanical forces at cellular level in the context of biological development.

The authors developed a top-down method for inferring pairwise potentials of cell-cell interactions driven by empirical evidence from 3D+time data of moving cells

The role of mechanical forces in shaping morphologies in biological development.

However, the paper is poorly written and might benefit from a re-write of sizable portions.

The authors do a nice work at circumscribing the problem, pose the problem very well

Fit for publication upon addressing the following concerns

# Writing

- Line 108: “However, mechanical parameters … remain …”
- Rephrase Lines 113-121: The end of the first sentence “as follows” suggests a list of actions. The next sentence breaks the flow. A potential following could be “Cell-cell adhesion forces, which are …”
- Line 118: Clarify “whereas both excluded volume effect …”
- Line 122: Did you mean “pairwise potentials of objects such as …”
- Figure 4F,G: Did you mean “cell-cell distance”?
- Lines 301-303: Are the potentials normalized by distances? If not, please rephrase.
- Line 426: “Our findings provide for the first time …”. Kindly consider [*https://doi.org/10.1101/2022.11.05.515310*](https://doi.org/10.1101/2022.11.05.515310)
- Line 458-459: “It remains unknown whether pairwise potentials can be …”. Kindly consider [*https://doi.org/10.1371/journal.pone.0254763*](https://doi.org/10.1371/journal.pone.0254763)*,* [*https://doi.org/10.1016/j.cels.2019.02.007*](https://doi.org/10.1016/j.cels.2019.02.007)
- Line 467-468: Did you mean “It is important … factors affect inferenced”

# Method

- Lines 144-145: “Our method was validated by using various synthetic data generated by simulations assuming cell proliferation”. How was this synthetic data simulated? Why not validate with a subset of the real dataset?
- How would you adapt the model and the cost function for non-constant physical conditions (e.g. \lambda variable)
- How does the method behave with simulated data obtained from different potentials (e.g. in addition to LJ, Morse potential, …)? Other than the visual appreciation of the resemblances between real and reconstructed DF curves, is there a quantitative metric (e.g. L2 norm)?
- Two cases were identified for which the method did not produce thrilling results: longer time intervals, and restricted space. Do these observations have any bearings on the overall validity of the method?
- How is division handled?

# Application in C. elegans and mouse

- Fig 4G: Could you comment on the slight deviations observed for stages t36-75 and t76-115. Are these deviations significant enough to infer different mechanical conditions in the embryo during these stages?
